# Female genital tract polyarteritis nodosa associated with cutaneous lymphocytic thrombophilic arteritis

**DOI:** 10.1093/rap/rkag056

**Published:** 2026-05-21

**Authors:** Abuelmagd Abdalla, Aoife Boyle, Mohamed Hag, Ian McDonald, Graham Woods

**Affiliations:** Rheumatology Department, Mater Misericordiae University Hospital, Dublin, Ireland; School of Medicine, University College Dublin, Belfield, Dublin, Ireland; Dermatology Department, Mater Misericordiae University Hospital, Dublin, Ireland; Infectious Diseases Department, Mater Misericordiae University Hospital, Dublin, Ireland; School of Medicine, University College Dublin, Belfield, Dublin, Ireland; Dermatology Department, Mater Misericordiae University Hospital, Dublin, Ireland; School of Medicine, University College Dublin, Belfield, Dublin, Ireland; Pathology Department, Mater Misericordiae University Hospital, Dublin, Ireland

Key message

- An LTA-pattern skin biopsy may indicate a broader PAN-spectrum process with internal organ involvement.

Dear Editor, Vasculitis of the female genital tract is uncommon. It has traditionally been seen as a local and often clinically benign process, especially when limited to the cervix [1, 2]. Gonadal involvement in men is a known manifestation of polyarteritis nodosa (PAN). However, the significance of vasculitis in the female genital tract with respect to systemic PAN is less clear. Cutaneous lymphocytic thrombophilic arteritis (LTA), also called macular lymphocytic arteritis (MLA), is generally considered an isolated mild skin arteritis without systemic involvement. Yet, its relationship to cutaneous PAN is debated. Some authors suggest that LTA may be part of the same clinicopathological spectrum, possibly representing a reparative or less necrotising phase of cutaneous PAN [3].

A 42-year-old woman was referred to our centre after incidental histological evidence of vasculitis was found 3 years earlier. This followed her hysterectomy with salpingo-oophorectomy for heavy fibroid bleeding. Histology revealed fibrinoid necrosis and chronic lymphocytic perivascular inflammation in vessels of the cervix, uterus and fallopian tubes (Fig. 1B). At the rheumatology and dermatology review, she was normotensive. She had a chronic livedoid eruption over both lower limbs, with milder involvement of the forearms (Fig. 1). Routine laboratory tests, autoantibody screening and angiographic assessment were normal. A 4-mm punch biopsy from her left thigh showed lymphocytic inflammation involving small and medium vessels in the deep dermis with thrombus formation, but no fibrinoid necrosis (Fig. 1A). This was consistent with cutaneous LTA. Re-evaluation of the earliest genital tract histology confirmed a similar vasculitis pattern affecting vessels of equivalent size, with a predominantly lymphocytic infiltrate. However, fibrinoid necrosis was only seen in the genital tract specimen. The results together supported PAN involving the female genital tract, along with cutaneous LTA-pattern arteritis, rather than two unrelated processes. She was treated with methotrexate and remained in remission at the 3-year follow-up.

**Figure 1:**
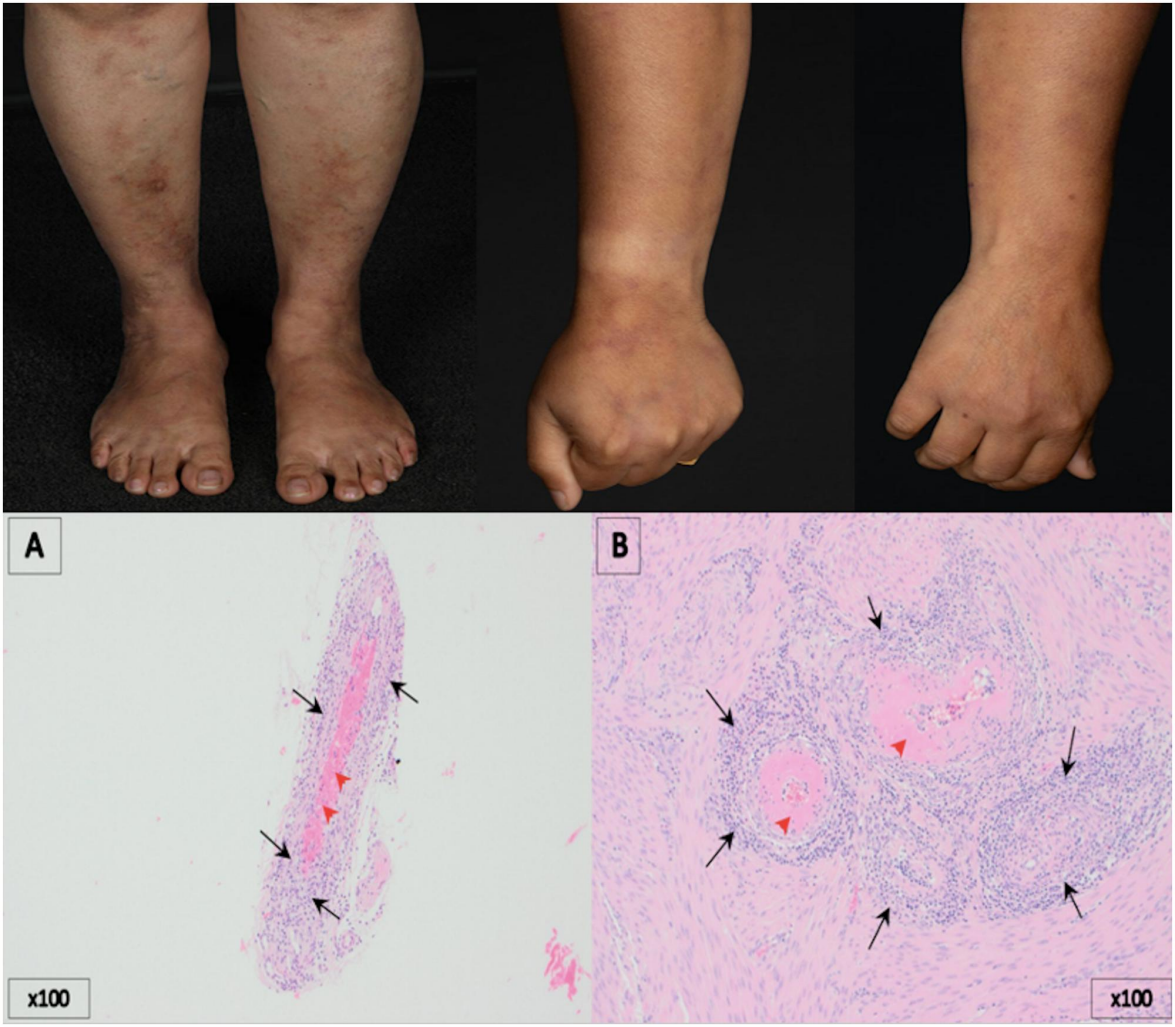
Upper panels: livedoid eruption affecting both lower limbs, with subtler livedoid change over the forearms. (A) Skin biopsy showing a medium-sized dermal vessel with lymphocytic inflammatory infiltrate (black arrows) and luminal thrombus formation (red arrowheads), without fibrinoid necrosis, consistent with cutaneous LTA. (B) Genital tract specimen within myometrium showing medium-sized arteries with lymphocytic inflammatory infiltrate (black arrows) and fibrinoid necrosis (red arrowheads). Original magnification ×100

Prior studies have shown that features considered characteristic of MLA/LTA may also be observed in cutaneous PAN, supporting overlap among these entities rather than strict separation [4]. These overlapping features include livedoid change, lymphocytic arteritis affecting medium-sized vessels and the absence of fibrinoid necrosis in more indolent lesions [5, 6]. Our case extends this discussion by demonstrating extracutaneous vasculitic involvement in association with an LTA-pattern skin biopsy. The combination of persistent livedoid cutaneous change and concordant histopathological findings in extracutaneous tissue should therefore prompt careful clinicopathological correlation and reconsideration of systemic PAN.

This case broadens the limited literature on female genital tract vasculitides, advances the debate on the relationship between LTA and PAN and illustrates that overlap between LTA and the PAN spectrum occurs, particularly when there is extracutaneous involvement, as both conditions may share clinical and histopathological features.

## Data Availability

The data underlying this article are not publicly available in order to protect patient confidentiality.

## References

[1] Ganesan R , Ferryman SR, Meier L et al Vasculitis of the female genital tract with clinicopathologic correlation: a study of 46 cases with follow-up. Int J Gynecol Pathol 2000;19:258–65. 10907175 10.1097/00004347-200007000-00010

[2] Hoppé E , de Ybarlucéa L-R, Collet J et al Isolated vasculitis of the female genital tract: a case series and review of literature. Virchows Arch 2007;451:1083–9. 17912548 10.1007/s00428-007-0514-4

[3] Macarenco RS , Galan A, Simoni PM et al Cutaneous lymphocytic thrombophilic (macular) arteritis: a distinct entity or an indolent (reparative) stage of cutaneous polyarteritis nodosa? Report of 2 cases of cutaneous arteritis and review of the literature. Am J Dermatopathol 2013;35:213–9. 22688396 10.1097/DAD.0b013e31825ba0ec

[4] Buffière-Morgado A , Battistella M, Vignon-Pennamen MD et al Relationship between cutaneous polyarteritis nodosa (cPAN) and macular lymphocytic arteritis (MLA): blinded histologic assessment of 35 cPAN cases. J Am Acad Dermatol 2015;73:1013–20. 26464220 10.1016/j.jaad.2015.09.010

[5] Morimoto A , Chen KR. Reappraisal of histopathology of cutaneous polyarteritis nodosa. J Cutan Pathol 2016;43:1131–8. 27592619 10.1111/cup.12809

[6] Kelly RI , Wee E, Balta S et al Lymphocytic thrombophilic arteritis and cutaneous polyarteritis nodosa: clinicopathologic comparison with blinded histologic assessment. J Am Acad Dermatol 2020;83:501–8. 32044177 10.1016/j.jaad.2019.10.068

